# The Association of PPARγ Pro12Ala and C161T Polymorphisms with Polycystic Ovary Syndrome and Their Influence on Lipid and Lipoprotein Profiles 

**DOI:** 10.22074/ijfs.2018.5270

**Published:** 2018-03-18

**Authors:** Zohreh Rahimi, Foroogh Chamaie-Nejad, Shohreh Saeidi, Ziba Rahimi, Ali Ebrahimi, Ebrahim Shakiba, Asad Vaisi-Raygani

**Affiliations:** 1 Medical Biology Research Center, Kermanshah University of Medical Sciences, Kermanshah, Iran; 2 Department of Clinical Biochemistry, Medical School, Kermanshah University of Medical Sciences, Kermanshah, Iran; 3 Department of Dermatology, Kermanshah University of Medical Sciences, Kermanshah, Iran; 4 Fertlility and Infertility Research Centre, Kermanshah University of Medical Sciences, Kermanshah, Iran

**Keywords:** Estradiol, Lipid, Lipoprotein, Peroxisome Proliferator-Activated Receptor, Polycystic Ovary Syndrome

## Abstract

**Background:**

The aim of present study was to clarify the role of the peroxisome proliferator-activated receptor (PPAR) γ Pro12Ala and C161T polymorphisms in the pathogenesis of polycystic ovary syndrome (PCOS) and their influence on lipid and lipoprotein profiles of patients.

**Materials and Methods:**

The present cross-sectional study consisted of 50 women with PCOS, who referred to the Kermanshah University of Medical Sciences Clinic between April and October 2015, and 233 unrelated age-matched healthy women from the same region (West Iran). The PPARγ Pro12Ala and PPARγ C161T polymorphisms were gen- otyped using the polymerase chain reaction-restriction fragment length polymorphism method. Fasting blood sugar (FBS), serum triglycerides (TG), cholesterol, low density lipoprotein- cholesterol (LDL-C), high density lipoprotein- cholesterol (HDL-C) and estradiol levels were measured.

**Results:**

The serum level of estradiol was significantly lower in PCOS patients compared to healthy women. The PPARγ Pro12Ala (CG) genotype increased the risk of PCOS 2.96-fold. The frequency of the PPARγ T allele (at C161T) was 21% in patients and 17.2% in controls with no significant difference (P=0.52). In all studied individuals, the PPARγ CG geno- type was associated with significantly higher levels of TG. However, significantly lower levels of total cholesterol and LDL-C were observed in PPARγ TT individuals compared with those with the CC genotype. Within the PCOS group, the PPARγ CG genotype was significantly associated with lower levels of estradiol compared with the CC genotype. Also, the CG genotype was significantly associated with higher levels of TG when compared with the CC genotype.

**Conclusion:**

Our study shows that, unlike PPARγ C161T, PPARγ Pro12Ala is associated with the risk of PCOS. Also, we found that the lipid and lipoprotein profiles significantly vary based on PPARγ Pro12Ala and C161T genotypes.

## Introduction

Polycystic ovary syndrome (PCOS) is one of the most frequent endocrine-related gynecological disorders among women of reproductive age (1). PCOS, a leading cause of female infertility, is characterized by hyperandrogenism, menstrual irregularity, chronic anovulation and multiple small sub-capsular ovarian cystic follicles (2). Around 50 to 70% of patients with PCOS are diagnosed with dyslipidemia (3).

The peroxisome proliferator-activated receptors (PPARs) belong to the nuclear hormone receptors that regulate the transcription of a variety of genes such as those involved in the metabolism of lipids in adipose tissue, liver and skin (4). The isoform PPARγ, which participates in lipid and glucose metabolism, is mainly expressed in adipose tissue (5).

The common *PPARγ* single nucleotide polymorphism (SNP) Pro12Ala (C/G; rs1801282) modulates its tran. scriptional activity, resulting in reduced transcriptional activity of PPARγ (4). The association of this SNPwith PCOS has been investigated, however, there are inconsistent reports about the role of this polymorphism in susceptibility to PCOS, and its influence on lipid and lipo. protein profiles (5-9).

The *PPARγ* SNP C161T(rs3856806, His447His) in exon 6 is also associated with decreased transcription of *PPARγ* (10). The role of this polymorphism in susceptibility to PCOS has also been studied but remains controversial (5, 8, 11, 12).

The aim of this study was to assess the association of *PPARγPro12Ala* and *C161T* variants with the risk of PCOS, and with lipid and lipoprotein profiles. In addition, we examined the association of both SNPs with the levels of estradiol and sex hormone binding globulin (SHBG) in a population from West Iran with a Kurdish ethnic background.

## Materials and Methods

The present cross-sectional study consisted of 50 women with confirmed PCOS according to the Rotterdam criteria (13), who referred to the Kermanshah University of Medical Sciences Clinic between April and October 2015. The mean age of PCOS women was 23.6 ± 5.3 years (ranging between 14 and 43 years). A total of 233 unrelated age-matched healthy individuals without PCOS were also included in this study with the mean age of 22.2 ± 4.2 years, (ranging between 18 and 33 years, P=0.09). Controls were volunteers from students and staff of Kermanshah University of Medical Sciences without any history of hyperandrogenism reflected by the presence of hirsutism, acne or alopecia and menstrual irregularity. 

Two out of three criteria of clinical and/or biochemical signs of PCOS, namely hyperandrogenism (the presence of hirsutism), acne or alopecia and ovarian dysfunction (oligo- and/or anovulation and/or polycystic ovaries detected by ultrasound scans) were sufficient to diagnose PCOS. Exclusion criteria were congenital adrenal hyperplasia, androgen-secreting tumors, and intake of any medication that may affect the endocrinal parameters along with the glucose and lipid profiles for at least 3 months prior to enrolment.

Height and weight were obtained from each individual and the body mass index (BMI) was calculated. All women in this study were from the Kermanshah province in West Iran, belonging to the Kurdish ethnicity.

All individuals agreed to participate in the study and signed a written informed consent before participation. The Ethics Committee of Kermanshah University of Medical Sciences approved the study. The study was in accordance with the principles of the Declaration of Helsinki II.

### Biochemical analysis

From each individual, a sample of 10 milliliters of venous blood was collected at 9 am under standard conditions. The sample was divided to two portions of six milliliters; portion one was centrifuged for 10 minutes at 1600 g in the absence of any anticoagulant and the obtained serum was used for biochemical analysis according to the standard protocol. The second portion (4 ml) was treated with EDTA and used for DNA extraction and further genetic analysis. 

The levels of fasting blood sugar (FBS), triglycerides (TG), cholesterol, low density lipoprotein-cholesterol (LDL-C) and high density lipoprotein-cholesterol (HDL- C) were measured using the Bionic Diagnostic Kits (Iran) on Mindray BS-480 Chemistry Analyzer (China). Serum estradiol level in the mid-follicular phase of the menstrual cycle and SHBG were measured using the chemiluminescent method by using the Abbott Architect i1000 (Abbott Laboratory, USA).

### Genotyping

DNA was extracted from venous blood using the standard phenol-chloroform method (14). The polymerase chain reaction (PCR)-restriction fragment length polymorphism (RFLP) was used to genotype the *PPARγ Pro12Ala* (C/G) SNP by using specific.

F: 5'-GCCAATTCAAGCCCAGTC-3'

R: 5'-GATATGTTTGCAGACAGTGTATCAGTGAAGGAATCGCTTTCCG- 3' primers.

**Fig.1:**
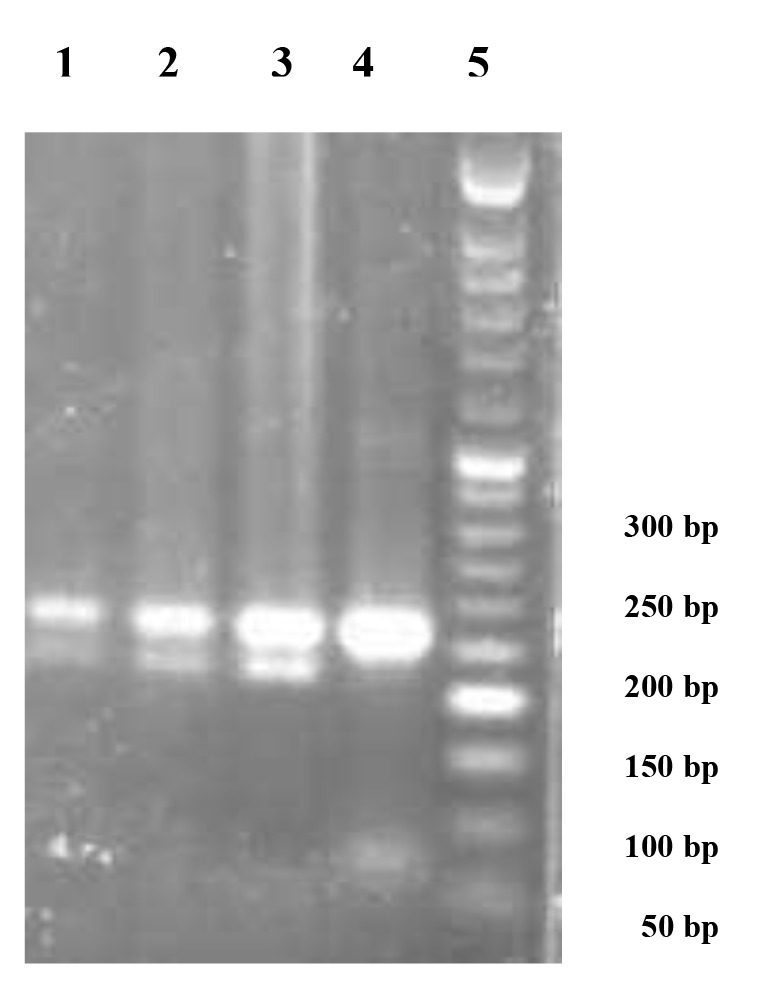
Agarose gel electrophoresis (2%) pattern of digested polymerase chain reaction (PCR) products by the BstU I restriction enzyme. From left to right, lanes 1, 2, and 3 represent the PPARγ CG genotype, lane 4 indicates the CC genotype and lane 5 shows the 50 bp DNA molecular weight marker.

The PCR reaction in a final volume of 25 µl contained 20 pmol of each primer, 100-200 ng DNA, 200 µM dNTPs, 1.5 mM MgCl2, 1 U Taq polymerase and 2.5 µl of 10X PCR buffer (SinaClon, Iran). The PCR conditions were an initial denaturation at 94°C for 5 minutes followed by 30 cycles of 94°C for 60 seconds, 55°C for 60 seconds and 72°C for 60 seconds, with a final extension for 5 minutes at 72°C. Five microliters of the resulting 270 bp PCR product was examined using electrophoresis on a 1% agarose gel containing the Gel Red (Kawsar Biotech Company, Iran) stain and was visualized under a UV Gel Documentation System (Quantum ST4). Fifteen microliters of the PCR product was treated with 5 U of the restriction enzyme BstU I at 37°C overnight and the RFLP products were electrophoresed on a 2% agarose gel (7). The C allele (ancestral) was not digested by the *BstU I* while the C to G substitution resulted in digestion of the PCR product into two fragments of 227 bp and 43 bp (Fig .1).

The PPARγ C161T SNP was detected by PCR-RFLP using specific

F: 5'-CAA GAC AAC CTG CTA CAA GC-3'

R: 5' -TCC TTG TAG ATC TCC TGC AG -3' primers.

**Fig.2:**
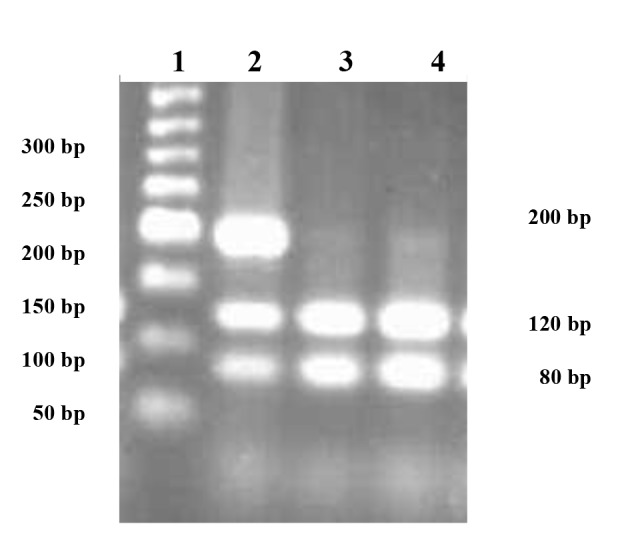
The agarose gel electrophoresis of restriction fragment length polymorphism (RFLP) products obtained by digestion of polymerase chain reaction (PCR) products by the *Pml1* restriction enzyme. From left to right, lanes 1, 2, and 3 and 4 represents the 50 bp DNA molecular weight marker, the CT genotype of *PPARγ C161T* and the wild type genotype of CC.

The PCR reaction consisted of 20 pmol of each primer, 100-200 ng DNA, 200 µM dNTPs, 1.5 mM MgCl2, 1 U Taq polymerase and 2.5 µl of 10X PCR buffer in a final volume of 25 µl. The PCR thermal cycling conditions were an initial denaturation at 94°C for 5 minutes, followed by 35 cycles by 94°C for 60 seconds, 55°C for 60 seconds and 72°C for 60 seconds, with a final extension for 5 minutes at 72°C. Five microliters of the resulting 200 bp PCR product was examined using electrophoresis on a 1% agarose gel containing Gel Red stain and visualized under a UV Gel Documentation System (Quantum ST4). Fifteen microliters of the PCR product were treated with 5 U of the restriction enzyme Pml1 at 37°C overnight and the RFLP products were electrophoresed on a 2% agarose gel (10). The ancestral allele fragment was digested into two fragments of 120 bp and 80 bp, while the derived allele remained intact (Fig .2).

### Statistical analysis

The frequency of alleles was calculated by the chromosome counting method and deviation from the Hardy-Weinberg equilibrium (HWE) was calculated using the Chi-square test. Comparison of genotype and allele frequencies of the two SNPs between PCOS patients and controls was undertaken using the Chi-square test. The SPSS logistic regression was used to calculate odds ratio (OR) as an estimate of relative risk for the disease and its 95% confidence interval (CI). The association between biochemical data and SNPs was calculated using the independent-sample t test and ANOVA. The P<0.05 was considered as statistically significant. The statistical package for social sciences (SPSS, SPSS Inc., Chicago, IL) version 16.0 was used for the statistical analysis.

## Results

Demographic and biochemical characteristics of the participants are presented in Table 1. Patients were age- matched with controls (P=0.09). Also, the two groups were BMI-matched (P=0.25, Table 1). A significantly lower serum level of estradiol was observed in PCOS women compared with controls (70 ± 45.5 vs. 109.7 ± 91.2 pg/ml respectively, P<0.001). However, a lower level of SHBG was observed in patients (52.2 ± 24.5) compared with controls (58.6 ± 33.9) but was not statistically significant (Table 1).

**Table 1:** Characteristics of PCOS patients and controls


Variable	Patient n=50 Mean ± SD	Control n=233 Mean ± SD	P value

Age (Y)	23.6 ± 5.3	22.2 ± 4.2	0.09
BMI (Kg/m^2^)	23.7 ± 4.9	22.8 ± 5.8	0.25
FBS (mg/dl)	78.6 ± 13.2	78.5 ± 14.8	0.97
Cholesterol (mg/dl)	131.1 ± 32.8	129.7 ± 30.6	0.78
TG (mg/dl)	78.8 ± 43.2	88 ± 51.5	0.25
HDL-C (mg/dl)	45.6 ± 11.7	46.5 ± 12.8	0.61
LDL-C (mg/dl)	74 ± 26.6	74.8 ± 24.5	0.82
Estradiol (pg/ml)	70 ± 45.5	109.7 ± 91.2	<0.001
SHBG (nmol/l)	52.2 ± 24.5	58.6 ± 33.9	0.13


PCOS; Polycystic ovary syndrome, BMI; Body mass index, FBS; Fasting blood sugar, TG; Triglycerides, HDL-C; High density lipoprotein-cholesterol, LDL-C; Low density lipoprotein-cholesterol, and SHBG; Sex hormone binding globulin.

The genotypic distribution of *PPARγ Pro12Ala* was in HWE in both patients and controls (P>0.1). However, the genotypic distribution of *PPARγ C161T* significantly deviated only in the control group (χ^2^=5.03, P<0.05). 

The genotype and allele frequencies of both SNPs are given in Tables 2, 3. The frequency of the CG genotype in patients was 32% and significantly higher than that in controls (13.7%, P=0.002, OR=2.96 (95% CI of 1.46-5.96) (Table 2). Given that the control group deviated from Hardy-Weinberg equilibrium for the C161T SNP, no further analysis was undertaken on the potential association of this SNP with PCOS.

**Table 2:** The frequency of PPARγ Pro12Ala (C/G) genotypes and alleles in patients and controls


Parameter		Patient n=50 (%)	Control n=233 (%)

Genotypes			
	CC	CC	201 (86.3)
	CG	CG	32 (13.7)
		χ^2^=9.75, P=0.002, OR=2.96 , (95% CI: 1.46-5.96, P=0.002)	
Alleles	Alleles	
	C	C	434 (93.1)
	G	G	32 (6.9)
		χ^2^=9.75, P=0.002, OR=2.96 (95% CI: 1.46-5.96, P=0.002)	


OR; Odds ratio and CI; Confidence interval.

The effect of both polymorphisms on lipid and lipoprotein profiles along with estradiol and SHBG levels in all studied individuals is shown in Table 4. A significantly higher level of TG was detected in the presence of the *PPARγ CG* (101.1 ± 59.4 mg/dl) genotype compared to the CC genotype (76.0 ± 40 mg/dl). Considering the effect of the *PPARγ C161T* polymorphism on lipid and lipoprotein profiles along with the estradiol level, we observed significantly lower levels of total cholesterol (85.5 ± 24.4 mg/dl, P=0.011) and LDL-C (42.5 ± 12.8 mg/dl, P=0.023) in homozygote TT individuals compared to those with the CC genotype (130.6 ± 30.6 and 75.4 ± 24.5 mg/dl respectively). The SHBG level was not significantly different between different genotypes of the two SNPs (Table 4).

**Table 3:** The genotype and allele frequencies of PPARγ C161T in the patient and control groups


Parameter		Patient n=50(%)	Control n=233(%)

Genotypes			
	CC	31 (62)	155 (66.5)
	CT	17 (34)	76 (32.6)
	TT	2 (4)	2 (0.9)
		χ^2^=3.05, P=0.21	
Alleles			
	C	79 (79)	386 (82.8)
	T	21 (21)	80 (17.2)
		χ^2^=0.4, P=0.52	


When each group was studied separately, the association of the *PPARγ CG* genotype, compared with the CC genotype, with significantly lower level of estradiol was only observed in the PCOS group (54.3 ± 28.9 pg/ml vs.77.9 ± 50.5 pg/ml, P=0.045). Also, a significantly higher level of TG was associated with the CG genotyped compared to the CC genotype (115.6 ± 62.4 and 74.6 ± 39.9 mg/dl respectively, P=0.026).

## Discussion

We identified an association between the PPARγ Pro12Ala CG genotype and the risk of PCOS in our population. We did not detect the GG genotype among our studied individuals because the homozygote Ala genotype is rare in the overall population (7).

There are inconsistent reports on the association of *PPARγ SNPs* with susceptibility to PCOS. This may be due to different frequencies of this SNP among different populations, but also different lifestyle, effects of environmental factors and also the influence of sample size.

In a study from Germany, the frequency of the *PPARγ Pro12Ala* SNP was not significantly different between PCOS and healthy women (7). Also, among Italians, the Pro12Ala SNP was unrelated to the risk of PCOS (5). However, among PCOS patients of Indian origin, the *PPARγ Pro12Ala* was associated with decreased PCOS susceptibility. However, the *PPARγ C161T *(His44His) did not affect the risk of PCOS among Indian (8) Caucasian (11) and Greek (12) women. In contrast, among the Italians, there was a significantly higher frequency of *PPARγ* T allele in PCOS patients than in controls (5). Meta-analysis by Zhang et al. (15) indicated that the Pro12Ala polymorphism reduced the risk of PCOS only in European but not in Asian populations.

**Table 4:** Mean number of primordial, primary, growing, atretic graafian follicles, graafian follicles and corpora lutea in the ovaries of rats in the experimental and control groups


Variable	*PPAR Pro12Ala (C/G)*		*PPAR C161T*			
	CC (n=235)	CG (n=48)	CC (n=186)	CT (n=93)	TT (n=4)

FBS (mg/dl)	78.7 ± 15	77.3 ± 11.6	79.4 ± 15.8	77.3 ± 11.3	63.3 ± 9.1
	P=0.61			P=0.47	P=0.06
Cholesterol (mg/dl)	129.0 ± 30.5	134.1 ± 32.9	130.6 ± 30.6	130.6 ± 30.7	85.5 ± 24.4
	P=0.33			P=1	P=0.011^*^
					P=0.012^**^
TG (mg/dl)	76.0 ± 40	101.1 ± 59.4	81.7 ± 46.4	79.8 ± 42.1	40.3 ± 18.4
	P=0.007			P=0.94	P=0.16
HDL-C (mg/dl)	46.8 ± 13	44.3 ± 10.6	46.9 ± 13.1	45.7 ± 11.7	36.0 ± 10.4
	P=0.16			P=0.71	P=0.2
LDL-C (mg/dl)	74.3 ± 24.4	76.1 ± 26.9	75.4 ± 24.5	74.5 ± 25.1	42.5 ± 12.8
	P=0.67			P=0.95	P=0.023^*^
					P=0.031^**^
Estradiol (pg/ml)	103.8 ± 86.6	96.8 ± 84.1	102.3 ± 86.5	103.6 ± 87	91.6 ± 57.2
	P=0.61			P=0.99	P=0.96
SHBG (nmol/l)	58.3 ± 33.8	53.2 ± 24.6	58.3 ± 34.2	55.2 ± 28.9	72.9 ± 32.1
	P=0.24			P=0.44	P=0.4


Data are presented as mean ± SD.*; Compared with the CC genotype, **; Compared with the CT genotype, FBS; Fasting blood sugar, TG; Triglycerides, HDL-C; High density lipoprotein-cholesterol, LDL-C; Low density lipoprotein-cholesterol, and SHBG; Sex hormone binding globulin

The PPARγ is a critical transcription factor involved in regulating glucose and lipid metabolism (16). The PPARγ is involved in energy regulation and fat deposition, and is recognized as an important gene contributing to obesity, obesity induced insulin resistance and dyslipidemia (8). The natural ligands of PPARs are unsaturated fatty acids, eicosanoids, oxidized LDL and VLDL, and linoleic acid derivatives. Fibrates and thiazolidinediones are pharmacological agonists of PPARs (17). Although we showed significant associations between the *PPARγ Pro12Ala* SNP and the lipid and lipoprotein profiles in a Kurdish population, this was not observed in a German population (7). Also, in a PCOS patient group of Italian origin, no significant difference in adiponectin, HDL-C, LDL-C and TG levels was observed between ancestral and variant genotypes of this SNP (5). In contrast, in PCOS women from Korea, a significantly increased HDL-C level was detected in individuals carrying the variant allele (9). The small sample size of the studied PCOS patient group is the main limitation of the present study which may affect the association observed between *PPARγ* genotypes and PCOS, lipid and lipoprotein profiles, and estradiol and SHBG levels.

## Conclusion

Our study showed an association between *PPARγ Pro12Ala* and the risk of PCOS while no influence of *PPARγ C161T* on susceptibility to PCOS was observed. Also, we found that the lipid and lipoprotein profiles are affected by the presence of *PPARγ Pro12Ala* and *C161T* polymorphisms. The ancestral CC genotype of C161T had a lowering effect on the TG level and the minor T allele had a beneficial effect in lowering cholesterol and LDL-C. In PCOS patients the variant CG genotype of Pro12Ala was associated with a lower level of estradiol and a higher concentration of TG.
